# Local Competition-Based Superpixel Segmentation Algorithm in Remote Sensing

**DOI:** 10.3390/s17061364

**Published:** 2017-06-12

**Authors:** Jiayin Liu, Zhenmin Tang, Ying Cui, Guoxing Wu

**Affiliations:** 1 School of Computer Science and Engineering, Nanjing University of Science and Technology, Nanjing 210094, China; tang.zm@mail.njust.edu.cn; 2 College of Computer Science and Engineering, Zhejiang University of Technology, Hangzhou 310023, China; cuiying@zjut.edu.cn; 3 Huawei Technologies Co., Ltd., Nanjing 210012, China; nt_wgx@126.com

**Keywords:** superpixel, remote sensing, local compete mechanism, boundary optimization, improved fast Gauss transform, fast marching method

## Abstract

Remote sensing technologies have been widely applied in urban environments’ monitoring, synthesis and modeling. Incorporating spatial information in perceptually coherent regions, superpixel-based approaches can effectively eliminate the “salt and pepper” phenomenon which is common in pixel-wise approaches. Compared with fixed-size windows, superpixels have adaptive sizes and shapes for different spatial structures. Moreover, superpixel-based algorithms can significantly improve computational efficiency owing to the greatly reduced number of image primitives. Hence, the superpixel algorithm, as a preprocessing technique, is more and more popularly used in remote sensing and many other fields. In this paper, we propose a superpixel segmentation algorithm called Superpixel Segmentation with Local Competition (SSLC), which utilizes a local competition mechanism to construct energy terms and label pixels. The local competition mechanism leads to energy terms locality and relativity, and thus, the proposed algorithm is less sensitive to the diversity of image content and scene layout. Consequently, SSLC could achieve consistent performance in different image regions. In addition, the Probability Density Function (PDF), which is estimated by Kernel Density Estimation (KDE) with the Gaussian kernel, is introduced to describe the color distribution of superpixels as a more sophisticated and accurate measure. To reduce computational complexity, a boundary optimization framework is introduced to only handle boundary pixels instead of the whole image. We conduct experiments to benchmark the proposed algorithm with the other state-of-the-art ones on the Berkeley Segmentation Dataset (BSD) and remote sensing images. Results demonstrate that the SSLC algorithm yields the best overall performance, while the computation time-efficiency is still competitive.

## 1. Introduction

Remote sensing technologies have been widely applied in urban environments’ monitoring, synthesis and modeling. However, the conventionally-used pixel-wise hyperspectral image (HSI) classification approaches [1,2] lack spatial information and thus may generate a “salt and pepper” result [3]. Fixed-size window-based HSI classification approaches [4,5] can extract local spatial information, but they cannot adaptively capture the structural characters of varying sizes and shapes [6]. Therefore, fixed-size window-based approaches may corrupt the result or result in some over-smoothing.

Superpixel [7] algorithms aim to over-segment an image into a configurable number of regions that are expected to be coherence in appearance and conform to the local image structure. Unlike pixels, superpixels incorporate the spatial structures of the image and the contextual information of pixels [8]. Compared with fixed-size square windows, superpixels have adaptive sizes and shapes for different spatial structures [9]. Moreover, by using hundreds of regions represent an image instead of $10^{5}$ pixels, it greatly reduces the complexity of consequent image processing tasks, especially for probabilistic, combinatorial or discriminative approaches.

With these advantages, superpixel segmentation has been introduced for remote sensing image processing, such as HSI compression [8], remote sensing image classification [3,6,9,10,11,12,13,14,15,16,17,18], HSI denoising [19] and target detection [20,21,22]. The watershed segmentation algorithm [23] is used for superpixel generation to enhance the discriminative power of the classifier [12,13]. N-Cut (Normalized Cut [24])-based classification is investigated to classify the Polarimetric Synthetic Aperture Radar (PolSAR) images by Liu et al. [14]. Fan et al. [19] integrate the Entropy Rate Superpixel (ERS) [25] into the Low-Rank Representation (LRR)-based denoising method to fully exploit both the spectral and spatial information of HSI, which led to better denoising quality. In the papers [6,9,10], they combine ERS and the first principal component of HSI to segment the HSI into K (number of superpixels) perceptually coherent regions, and then present a superpixel-based sparse model to exploit the spatial correlation between the superpixels for classification of the hyperspectral images. SLIC [26] has also been extended to the HSI for improving the classification performance [15,16,17,18] and target detection performance [20,21,22]. These studies show that the conventional superpixel algorithms or their extended versions have the potential to improve the remote sensing image processing performance. Besides, superpixel algorithms have also been widely adopted as a precursor to other various higher level processing tasks such as image segmentation [27], contour detection [28], road detection [29,30,31], image labeling [32], object tracking [33] and object localization [34].

**Figure 1:**
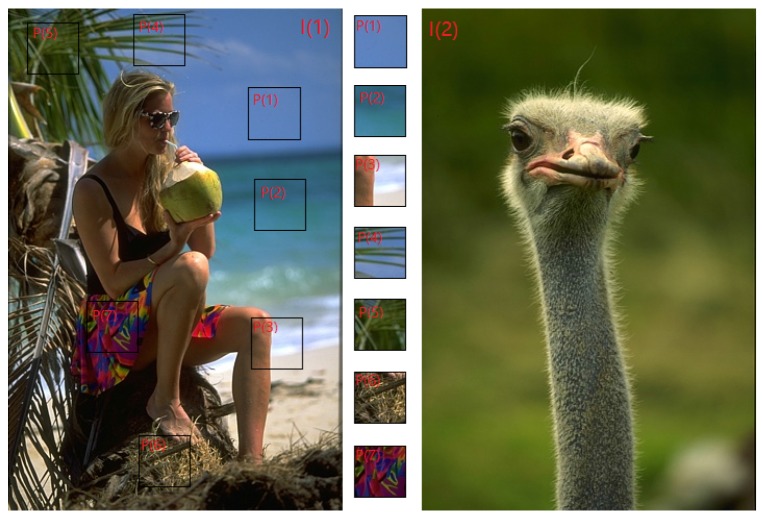
Image patches (from P(1) to P(7) in image I(1)) have diverse color and density layout and the same between I(1) and I(2).

An essential part of the conventional superpixel algorithms is the energy function and the corresponding optimization scheme adapted to it. Typically, an energy function has two or more energy terms, which describe the pixel-superpixel relationship. The frequently-used energy terms include the appearance coherence term on color space and the compactness constraint term on metric space. Ideally, to produce robust and consistent output, an energy term should be insensitive to the diversity of image content and scene layout. Major existing superpixel algorithms directly calculate energy terms in raw feature space with each term having a fixed weight regardless of heterogeneous scene layouts. Consequently, energy terms will function inconsistently between image regions (such as the appearance coherence term in SPixel [35], which is the Euclidean distance between the color of a pixel and the mean color of a superpixel, generates a larger energy value in regions with high color variance; therefore, it has a larger impact than in regions with low color variance). Meanwhile, as the image contents and scene layouts in different images vary largely, e.g., as shown in Figure 1, it seems impossible to seek a fixed set of optimal weight parameters for energy terms adaptable to every image.

For robustness and consistency, energy terms should be able to adapt to various image contents and scene layouts in arbitrary image regions. With improved consistency, energy terms can be used to optimize the weight parameters so that the energy function will further enhance its performance. For these purposes, a local competition superpixel segmentation algorithm is proposed that utilizes the competition mechanism in the following two levels:

- On pixel labeling level, by utilizing the competition mechanism in maximizing the energy function, each pixel gets a label.
- On energy term construction level, by utilizing the competition mechanism, which generates the membership degree of pixel-superpixel from raw measurements (where those raw measurements are derived from the original feature space, such as the lab color space in SLIC), the value of energy terms can be obtained.

Since each competition is restricted to a small local region, the proposed algorithm is named the Superpixel Segmentation with Local Competition (SSLC). The local competition mechanism leads to energy terms locality and relativity. Normally, changes of the image content and scene layout are small in nearby regions. Thus, the proposed algorithm is less sensitive to changes in image content and scene layout and performs much better in consistency across image regions.

Color appearance is one of the most important features for most state-of-the-art superpixel algorithms. SLIC [26] and SPixel [35] use the mean value to represent the color feature of a superpixel. The main disadvantage of this model is its failure to describe the color distribution, and the mean color bias can be large. To avoid these flaws, Lattice Cut [36] and Seeds [37] utilize the histogram describing the color distribution of the superpixel. However, histograms are not smooth, and their shapes depend on both the start points of bins and the width of bins. In order to avoid the aforementioned flaws, The KDE-estimated PDF is introduced to describe the color distribution of superpixels. This is a sophisticated measure that has the potential to describe the color distribution much more accurately, especially when the color distribution is diverse.

Recently, Spixel [35] and Seeds [37] introduced a boundary optimization scheme to generate superpixels. They show competitive efficiency and performance to the state-of-the-art ones. Nevertheless, both of these algorithms start from regular lattice grids, which is far from the optimal solution. Hence, the fast SLIC is adopted to generate initial partitions, which have a more coherent appearance and better boundary adherence. As a result, they promote the final result to converge to a more optimal solution.

We evaluated our approach on the berkeley segmentation dataset (BSD) [38]. Experimental results show that the proposed approach achieves higher boundary recall and achievable segmentation accuracy, as well as lower under-segmentation error than state-of-the-art superpixel segmentation algorithms. At the same time, it maintains competitive time efficiency. Therefore, the proposed algorithm is a good alternative method for remote sensing image processing.

The rest of this paper is organized as follows. Section 2 briefly reviews the existing techniques used for superpixel segmentation. Section 3 presents the proposed Superpixel Segmentation with Local Competition (SSLC) algorithm, followed by the detailed implementation of the proposed algorithm in Section 4. Experimental results are presented and analyzed in Section 5. We conclude this paper and plan for the future work in the final section.

## 2. Related Works

The term “superpixel” dates back to 1980s by simply regarding it as an image over-segmentation. While many traditional image segmentation algorithms (such as watershed [23], meanshift91 [39], FH [40], Quick Shift (QS) [41]) still work, those “general purpose” algorithms are not flexible enough to directly control the number of superpixels with a lack of compactness constraint. Superpixels produced by these traditional algorithms usually have highly irregular shapes and sizes and thus are no longer as comparable with “pixels”. Moreover, a large superpixel with a highly irregular shape tends to overlap with multiple objects.

The concept of superpixel algorithms was first introduced by Ren and Malik [7]. They use contour and texture cues to recursively partition a graph of all pixels in the image and globally minimize a cost function defined on the edges at the partition boundaries. Therefore, it produces superpixels with similar sizes and in a more compact shape. However, the superpixel segmentation algorithm in [7] has very high computational complexity and relatively poor boundary adherence performance.

Following that, a wide variety of superpixel algorithms has been proposed. Levinshtein et al. introduced an alternative algorithm named TurboPixel [42]. It places initial seeds regularly onto an image, and uses the level-set method to dilate progressively from those seeds based on local image gradients. The algorithm yields a lattice-like structure of compact superpixels with approximately uniform size. TurboPixel shows far better efficiency than the algorithm proposed by Ren and Malik. A variation of TurboPixel is presented by Peng et al. [43] where they invent a structure-sensitive over-segmentation technique, by over-segmenting images into regions with quasi-uniform density. However, both algorithms may fail when segmenting an image in regions with high intensity variability which leads to their results having poor performance in boundary adherence and under-segmentation error.

Some interesting works on superpixel algorithms are presented by Moore et al [36,44,45]. Their goal is somewhat different from the aforementioned methods. They seek to compute regular superpixels while preserving the topology. Superpixel Lattices [44] uses a greedy algorithm to cut images along vertical and horizontal strips sequentially. Consequently, superpixels that are produced by Superpixel Lattices preserve the inherent topology structure; whereas in Lattice Cut [36] the problem is solved with a graph-cut algorithm. It is a more global optimization, resulting in generating superpixels with better performance across all superpixel resolutions. Compared with lattice approaches, TPS [45] adopts local optimal constraints and offers better boundary recall than SP-Lattice. The major drawback of those algorithms is their performance depending on the quality of the pre-computed boundary maps.

Another widely-known superpixel algorithm, named simple linear iterative clustering (SLIC [26]), is proposed by Achanta et al. It is based on an iterative k-means clustering, and the similarity measure is the Euclidean distance in a three-dimensional color space, as well as two-dimensional image metric space. Despite its simplicity, SLIC shows superior performance and effectiveness over the existing methods at that time. However, it has one major drawback: the k-means algorithm does not guarantee the connection of all clusters, which is essential for superpixels. Therefore, a post-processing step is required to reconnect superpixels that have been ripped apart, thereby bypassing the distance measure. Based on this work, Li et al. propose Linear Spectral Clustering (LSC [46]). They first map each pixel to a ten-dimensional feature space, then apply a weighted k-means clustering to segment an image into a specified number of superpixles. It achieves better boundary adherence and is able to preserve the global properties of images. Another strategy for improving the performance is introduced by Liu et al. called Manifold SLIC [47]. Other than the conventional SLIC method that clusters pixels in $R^{5}$, they map the input image I onto a two-dimensional manifold *M* embedded in the combined image and color space $R^{5}$. The area elements of *M* reflect the density of image content. Through computing Restricted Centroidal Voronoi Tessellation (RCVT) on *M*, the manifold SLIC generates content-sensitive superpixels. Wang et al. [48] initialize cluster centers in a hexagon distribution rather than a square distribution and incorporate the boundary term into the distance measurement during k-means clustering. The generated superpixels by their approaches are shaped into regular and compact hexagons.

In contrast to the graph-cut-based methods and traditional gradient-ascent-based growing superpixels, Seeds [37] introduces a boundary optimization scheme to generate superpixels. It starts from regular grids, then iteratively optimizes them by moving the boundaries of the superpixels. By optimizing only the boundary pixel (or block) instead of the whole image, the computational complexity could be significantly decreased. Inspired by the Seeds algorithm, Spixel [35] uses a coarse-to-fine energy update strategy to optimize the boundary blocks (or pixels), which allows the optimization to reach better energy minima.

## 3. Problem Formulation via Local Competition Mechanism

### 3.1. Energy Function Formulation

Image segmentation can be represented by referring to the set of pixels in a superpixel:

(1) $$R_{k}=\left\{x_{i}:l_{x_{i}}=k\right\}$$

where set $R_{k}$ is identified by an integer label k(k∈L), *L* is a finite set of labels ($L=\left\{1,2,\cdots ,K\right\}$, *K* is the number of labels). $I={\left\{x_{i}\right\}}_{i=1}^{N}$ (*N* is the number of pixels in the image) denotes the set of all pixels in an image. $l_{x_{i}}$ denotes the label of pixel $x_{i}$. In superpixel segmentation, which is a case of image segmentation, the set $R_{k}$ is called superpixel $R_{k}$. According to Equation (1), when pixel $x_{i}$ belonging to superpixel $R_{k}$ or superpixel $R_{k}$ contains pixel $x_{i}$, it is equivalent to assigning label *k* to pixel $x_{i}$, i.e., $l_{x_{i}}=k$. Since a pixel only belongs to a single superpixel, it is a multiple-to-one mapping. Therefore, the set of superpixels is restricted to be disjoint; the intersection between any pair of superpixels is always an empty set: $R_{k}\cap R_{k^{\prime }}=\emptyset ,k\neq k^{\prime }$. Accordingly, the whole partition can be represented as $ℜ=\left\{R_{1},R_{2},\cdots ,R_{K}\right\}$.

The segmentation also can be considered as a labeling problem, i.e., assigning a unique label to each pixel. The full partitioning of an image can be represented with the set $\omega =\left\{l_{x_{1}},l_{x_{2}},\cdots ,l_{x_{N}}\right\}$. Superpixel algorithms are designed to find the best partition ω∈Ω, where Ω is denoted as the set of all partitions. This can be reached by maximizing an objective function, or so-called energy function, which is denoted as $E\left(\omega \right)$. Then, the partition that maximizes the energy function is defined as $\hat{\omega }$:

(2) $$\hat{\omega }=\arg\max_{\omega \in \Omega }E\left(\omega \right)$$

Let us review the properties that an ideal superpixel algorithm should have: (1) a properly connected group of pixels, which are exactly in one “semantic” region; (2) good adherence to the image boundaries; (3) the property of being as regular as possible for features that need spatial support. Given the above requirements, the energy function we have constructed integrates an appearance coherence term, a shape regularization term, a smooth constraints term and a connectivity term. Mathematically, it is formulated as:

(3) $$\begin{array}{c} E\left(\omega \right)=\lambda_{color}\sum_{x_{i}\in I}E_{color}\left(x_{i},l_{x_{i}}\right)+\lambda_{dist}\sum_{x_{i}\in I}E_{dist}\left(x_{i},l_{x_{i}}\right)\\ \;\;\;\;\;\;\;\;\;\;\;+\;\lambda_{smooth}\sum_{x_{i}\in I}E_{smooth}\left(x_{i},l_{x_{i}}\right)+\sum_{x_{i}\in I}E_{cnct}\left(x_{i},l_{x_{i}}\right) \end{array}$$

where $\lambda_{color}$, $\lambda_{dist}$, $\lambda_{smooth}$ are weight parameters. By adjusting the value of these parameters, the algorithm will produce different superpixel segmentations to meet the needs in different application scenarios. The greater the value of $\lambda_{color}$, the more appearance coherence is emphasized which usually lead to better boundary adherence. The greater the value of $\lambda_{dist}$ and $\lambda_{smooth}$, the more shape compactness is emphasized which usually lead to the generated superpixels with more regular shapes. The value of $\lambda_{color}$, $\lambda_{dist}$ and $\lambda_{smooth}$ can be in the range [1, 6], [1, 6] and [1, 2] respectively. We choose $\lambda_{color}=6,\lambda_{dist}=1$ and $\lambda_{smooth}=1$ as default parameters for the result of this paper.

### 3.2. Energy Terms Based on Local Competition Mechanism

In our algorithm, energy terms are not raw measurements, but a degree of pixel-superpixel membership, which is computed by the following steps.

1. Raw measurements are directly calculated in the original feature space, such as color similarity in the Lab color space and geodesic distances in image metric space. Given a pixel $x_{i}$, only the raw measurements between $x_{i}$ and each superpixel in the corresponding set $\Upsilon_{x_{i}}$ are computed; where $\Upsilon_{x_{i}}$ denotes the set of superpixels to which pixel $x_{i}$ may belong. $\Upsilon_{x_{i}}$ is a subset of *ℜ* and is restricted to a small local image region.
2. Energy terms are calculated by utilizing the local competition mechanism. This mechanism generates a membership degree of pixel-superpixel from raw measurements, which are computed in Step 1. The membership degrees are the value of energy terms. The energy term such as the appearance coherence term $E_{color}\left(x_{i},l_{x_{i}}\right)$ is generated from color similarity; the shape regularization term $E_{dist}\left(x_{i},l_{x_{i}}\right)$ is generated from geodesic distances.

This approach has the following advantages: (1) energy terms are membership degrees of the pixels and their corresponding superpixels, where these membership degrees are generated by local normalized the raw measurements in each feature space. Therefore, energy terms are irrelevant to the measurement units of feature spaces, such as color space, metric space, etc. (2) each competition is restricted to a small image region, generally the change of scene layout is small and features are consistency in small region. Therefore, energy terms, which are relative values, exhibit much more consistent performance and are less sensitive to the changes of scene layout.

#### 3.2.1. Appearance Coherence Term: $E_{color}\left(x_{i},l_{x_{i}}\right)$

**Figure 2:**
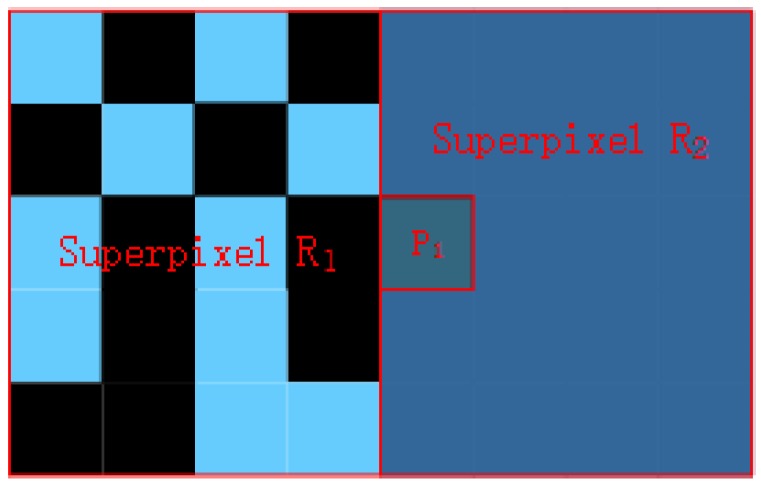
Disadvantage of using mean color to describe the color distribution of superpixels. Superpixel $R_{1}$ is composed of pixels whose RGB is (0,0,0) or (102,204,254), and the mean color is (51,102,127). The RGB of pixels in superpixel $R_{2}$ is (51,102,153). The RGB of pixels in region $P_{1}$ is (51,102,127). Red lines are boundaries of superpixels or regions.

Papers on superpixels introduce various energy terms to encourage generating superpixels with a perceptually-consistent appearance. The core of these terms is the manner in which they describe the color distribution of superpixels. SLIC [26] and SPixel [35] use the mean color to represent a superpixel’s color feature. The limitation of using the mean color is its failure to describe the color distribution, and the bias might be large. As shown in Figure 2, the mean color of superpixel $R_{1}$ equals the color of pixels in region $P_{1}$. Thus, the color energy term, such as in SLIC, which is calculated between pixels in region $P_{1}$ and superpixel $R_{1}$, is smaller than the one between them and superpixel $R_{2}$. This can cause the pixels in region $P_{1}$ to fall into superpixel $R_{1}$. This violates the intuitive consistency with the human vision system and produces a non-coherence over-segmentation result.

**Figure 3:**
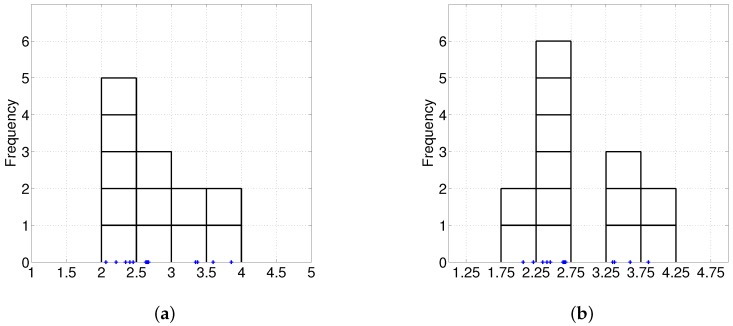
Effect of the start point in the histogram. (**a**) Start point = 0, bin width = 0.5; (**b**) Start point = 0.25, bin width = 0.5.

To avoid these flaws, Lattice Cut [36] utilizes a histogram to describe the color distribution of a superpixel. It quantizes the RGB values into a regular 3D-grid of 10×10×10 bins, forming a 1000D histogram at each superpixel. However, the histogram has inherent drawbacks. It is not smooth and its shape depends on the starting points of the bins (see in Figure 3) and the width of the bins.

In order to avoid the aforementioned flaws, a KDE-estimated PDF is introduced to describe the color distribution of superpixels. It is a sophisticated measurement that has the potential to describe color distribution much more accurately, especially when the color distribution is diverse. The color distribution $P_{R_{k}}\left(c\right)$ of superpixel $R_{k}$ is described as:

(4) $$P_{R_{k}}\left(c\right)=\frac{\sum_{x_{i}\in R_{k}}\phi_{c,c_{x_{i}}}}{n_{k}}$$

where $n_{k}$ denotes the number of pixel in $R_{k}$, *c* is a color vector and $c_{x_{i}}$ is the color vector of pixel $x_{i}$. ϕ is the kernel function.

Here, the Gaussian kernel is adopted to estimate PDF, then $P_{R_{k}}\left(c\right)$ can be represented as:

(5) $$P_{R_{k}}\left(c\right)=\frac{\sum_{x_{i}\in R_{k}}e^{-\frac{d^{2}\left(c,c_{x_{i}}\right)}{{\sigma_{k}}^{2}}}}{n_{k}\sqrt{2\pi }\sigma_{k}}$$

where $\sigma_{k}$ is the bandwidth of the Gaussian kernel. The weighted Euclidean distance between two color vector $d(c,c_{x_{i}})$ is defined as:

(6) $$d\left(c,c_{x_{i}}\right)=\sqrt{\sum_{j=1}^{d}w_{j}{\left(c^{j}-{c_{x_{i}}}^{j}\right)}^{2}}$$

where *d* is the dimension of the color vector (for color image, the color vector is in Lab color space; for hyperspectral image, the color vector is in original metric space) and $w_{j}$ is the weight of corresponding dimension in the color vector (the weights can be specified according to the prior knowledge of the data. Normally, such as for the color image, the weight of each dimension is one). The weighted Euclidean distance will be more accurate than the normal Euclidean distance when the effect of dimensions in color vector is different. In addition, the weighted Euclidean distance can be reduced as the normal Euclidean distance, for the case of those weights that have the same values.

As shown in Figure 1, the color distribution varies in different parts of an image, and in different images. This causes bandwidth requirements to vary widely in heterogeneous regions. For example, in Figure 1, the color variation is very small throughout much of the image I(2); the PDFs of superpixels in I(2) require a small bandwidth to distinguish the subtle color differences. In contrast, the left half of the image I(1) shows much higher color variance. Superpixel PDF in that region requires a larger bandwidth to diffuse the probability density in a larger neighborhood range. Thereby, they can effectively estimate the similarity in a larger color range. Based on such requirements, the bandwidth is defined as:

(7) $$\sigma_{k}=\left\{\begin{array}{c} s_{k}+\nu_{min}\;\;\;if\;\left(s_{k}+\nu_{min}\right)<\nu_{max}\\ \nu_{max}\;\;\;\;\;\;\;\;\;\;otherwise \end{array}\right.$$

$$s_{k}=\sqrt{\frac{\sum_{x_{i}\in R_{k}}d^{2}\left(c_{x_{i}},{\bar{c}}_{k}\right)}{n_{k}}}.$$

where ${\bar{c}}_{k},s_{k}$ are the mean color vector and standard deviation of superpixel $R_{k}$, respectively. Constant $\nu_{min}$ ($\nu_{min}=2$ in the experiment) avoids too small bandwidth situations. This sort of limited bandwidth results in a probability density distribution that concentrates around the observed data. On the contrary, $\nu_{max}$ ($\nu_{max}=16$ in the experiment) prevents the situation of too large bandwidth, which can lead to a probability density that is diffused over an excessive region and has the property of being relatively smooth. Thus, colors that are too close to each other or too different from each other cannot be well differentiated.

The computational complexity of directly estimating the discrete Gauss transform requires O(MN) operations (M is the number of points and N is the number of Gaussian kernel functions). In order to improve the computational efficiency, the Improved Fast Gauss Transform [49] (IFGT) is introduced to estimate PDF. Then, Equation (5) can be rewritten as:

(8) PRk(c)=∑m=1M∑α≤p−1Cαmed2(c,c¯m)σk2(w(c−c¯m)σk)α

(9) Cαm=2αα!∑xi∈Sme−cxi−c¯m2σk2(w(cxi−c¯m)σk)α

Here, an adaptive space partitioning scheme (the farthest point clustering algorithm) is introduced to divide $R_{k}$ into *M* (M=10 in the experiment) clusters ${\left\{S_{m}\right\}}_{m=1}^{M}$. ${\bar{c}}_{m}$ is the mean color vector of cluster $S_{m}$. Multi-index $\alpha =(\alpha_{1},\alpha_{2},\cdots ,\alpha_{d})$ is a d-tuple of nonnegative integers, the length of the multi-index α is defined as $\left|\alpha \right|=\alpha_{1}+\alpha_{2}+\cdots +\alpha_{d}$; the factorial of α! is defined as $\alpha !=\alpha_{1}!\alpha_{2}!\cdots \alpha_{d}!$. For any multi-index $\alpha \in N^{d}$ and $c\in R^{d}$ the d-variate monomial $c^{\alpha }$ is defined as $c^{\alpha }={c_{1}}^{\alpha_{1}}{c_{2}}^{\alpha_{2}}\cdots {c_{d}}^{\alpha_{d}}$. Each of the Taylor series are truncated after *p* (p=3 in the experiment) terms.

Through applying IFGT, estimating PDF only requires the computation of several multivariate polynomials instead of accumulative Gaussian function for each pixel in a superpixel. This reduces the computational complexity from quadratic order to linear order and makes the computational process optimal, such that it requires the minimal memory.

Once the color PDF of the superpixel is obtained, computing the pixel-superpixel color similarity is a straightforward task. As mentioned before, superpixels represent small regions with perceptually coherent appearance. Therefore, the greater the color similarity of a pixel-superpixel pair, the larger the value that should be produced by the appearance coherence term. We utilize the local competition mechanism to construct the appearance coherence term. This mechanism is implemented as follows: given a pixel $x_{i}$, we calculate color similarities between $x_{i}$ and each superpixel $R_{n}$ in set $\Upsilon_{x_{i}}$ and then normalize these color similarities. The normalized value is the membership degree of the pixel and its corresponding superpixel. Accordingly, the appearance coherence term $E_{color}\left(x_{i},l_{x_{i}}\right)$ is defined as:

(10) $$E_{color}\left(x_{i},l_{x_{i}}\right)=\left\{\begin{array}{c} \frac{P_{R_{l_{x_{i}}}}\left(c_{x_{i}}\right)}{\sum_{R_{n}\in \Upsilon_{x_{i}}}P_{R_{n}}\left(c_{x_{i}}\right)}\;\;\;if\;R_{l_{x_{i}}}\in \Upsilon_{x_{i}}\\ 0\;\;\;\;\;\;\;\;\;\;\;\;\;\;\;\;\;\;\;\;\;otherwise \end{array}\right.$$

#### 3.2.2. Shape Regularization Term: $E_{dist}\left(x_{i},l_{x_{i}}\right)$

As mentioned before, superpixels should be as regular as possible. Accordingly, a shape regularization term is constructed to penalize large space distance.

A commonly-used space distance is the Euclidean distance. As mentioned in [43], Euclidean distance is irrelevant to the image contents in-between. This leads to a measure that remains the same no matter whether there is a path along which the appearance transits smoothly. Nevertheless, geodesic distance has no such problem. Therefore, we use the geodesic distance [50] $D_{g}\left(u_{x_{i}},R_{k}\right)$ to measure the space distance between pixel $x_{i}$ and superpixel $R_{k}$:

(11) $$D_{g}\left(u_{x_{i}},R_{k}\right)=\min_{F_{u_{x_{i}},{\bar{u}}_{k}}}\int_{0}^{1}U\left(F_{u_{x_{i}},{\bar{u}}_{k}}\left(t\right)\right)∥{\dot{F}}_{u_{x_{i}},{\bar{u}}_{k}}\left(t\right)∥dt$$

where $u_{x_{i}}$ is the position of pixel $x_{i}$ and ${\bar{u}}_{k}$ is the centroid position of superpixel $R_{k}$; $F_{u_{x_{i}},{\bar{u}}_{k}}\left(t\right)$ is a path connecting the pixel $x_{i}$,${\bar{u}}_{k}$ (for t=0 and t=1 respectively). The gradient function U(x) is used as the distance increment, and $U\left(x\right)=G\left(x\right)+\kappa_{g}$, where G(x) is the gradient magnitude of the image. $\kappa_{g}$ ($\kappa_{g}=1$ in the experiment) is a constant that ensures that U(x) produces a constant distance increment (i.e., $U\left(x\right)=\kappa_{g}$ if G(x)=0) in absolutely consistent appearance regions and, thus, retains the minimum possible isoperimetric ratio. This ensures that the superpixels are compact and avoids large under-segmentation. We adopt the fast marching algorithm introduced by Sethian in [51] to compute geodesic distances for better computational efficiency.

In order to promote the generated superpixels compactness, the shape regularization term is used to penalize large space distance. Therefore, the smaller the geodesic distances of a pixel-superpixel pair, the larger the value should be produced by the shape regularization term. We utilize the local competition mechanism to construct the shape regularization term. This mechanism is implemented by the following: given a pixel $x_{i}$, we calculate geodesic distances between $x_{i}$ and each superpixel in set $\Upsilon_{x_{i}}$, and then, we generate the degree of pixel-superpixel membership by utilizing Equation (12) to normalize those geodesic distances:

(12) $$E_{dist}\left(x_{i},l_{x_{i}}\right)=\left\{\begin{array}{c} \frac{\sum_{R_{n}\in \Upsilon_{x_{i}}}D_{g}\left(u_{x_{i}},R_{n}\right)-D_{g}\left(u_{x_{i}},R_{l_{x_{i}}}\right)}{\left(\left|\Upsilon_{x_{i}}\right|-1\right)\sum_{R_{n}\in \Upsilon_{x_{i}}}D_{g}\left(u_{x_{i}},R_{n}\right)}\;\;\;\;\;if\;R_{l_{x_{i}}}\in \Upsilon_{x_{i}}\\ 0\;\;\;\;\;\;\;\;\;\;\;\;\;\;\;\;\;\;\;\;\;\;\;\;\;\;\;\;\;\;\;\;\;\;\;\;\;\;\;\;\;\;\;otherwise \end{array}\right.$$

where $\left|\cdot \right|$ denotes the size of a set.

Additionally, it can be observed that $D_{g}\left(u_{x_{i}},R_{k}\right)$ is a monotonically increasing function that is large on the edges. The geodesic distance of a path across an intensity boundary is always larger than that in the homogeneous region. Thus, it inherently promotes partitions adhering to image boundaries.

#### 3.2.3. Smoothness Term: $E_{smooth}\left(x_{i},l_{x_{i}}\right)$

The smoothness term promotes adjacent pixels, especially for the directly connect neighbor pixels, having the same label.

Firstly, we introduce a weighted chessboard distance. It is computed over the second-order eight neighborhood of pixel $x_{i}$ (we denote it as $\delta_{x_{i}}^{8}$).

(13) $$D_{s}\left(x_{i},R_{k}\right)=\lambda_{1}\sum_{x_{j}\in \left(\delta_{x_{i}}^{4}⋂R_{k}\right)}V(x_{i},x_{j})+\lambda_{2}\sum_{x_{j}\in \left(\left(\delta_{x_{i}}^{8}-\delta_{x_{i}}^{4}\right)⋂R_{k}\right)}V(x_{i},x_{j})$$

where $\delta_{x_{i}}^{4}$ is pixels within first-order four neighborhood of pixel $x_{i}$. Constant $\lambda_{1},\lambda_{2}$ ($\lambda_{1}\;=\;0.6,\lambda_{2}\;=\;0.424$ in the experiment) are weight parameters, which measure the impacts of directly adjacent pixels and diagonal pixels, respectively, $V(x_{i},x_{j})$ is an indicator variable, which is equal to one when $x_{i}$ and $x_{j}$ have the same label, i.e.,

(14) $$V\left(x_{i},x_{j}\right)=\left\{\begin{array}{c} 1\;\;\;\;\;if\;\;l_{x_{i}}=l_{x_{j}}\\ 0\;\;\;\;\;otherwise \end{array}\right.$$

The weighted chessboard distance facilitates the generated superpixels with smooth boundaries. By utilizing the local competition mechanism, the larger the weighted chessboard distance of a pixel-superpixel pair, the larger the value that should be produced by the smoothness term. Then, the smoothness term $E_{smooth}\left(x_{i},l_{x_{i}}\right)$ is defined as:

(15) $$E_{smooth}\left(x_{i},l_{x_{i}}\right)=\left\{\begin{array}{c} \frac{D_{s}\left(x_{i},R_{l_{x_{i}}}\right)}{\sum_{R_{n}\in \Upsilon_{x_{i}}}D_{s}\left(x_{i},R_{n}\right)}\;\;\;\;\;if\;R_{l_{x_{i}}}\in \Upsilon_{x_{i}}\\ 0\;\;\;\;\;\;\;\;\;\;\;\;\;\;\;\;\;\;\;\;\;\;\;\;\;otherwise \end{array}\right.$$

#### 3.2.4. Connectivity Term: $E_{cnct}\left(x_{i},l_{x_{i}}\right)$

It is essential for superpixels that pixels in a superpixel should be connected. Thus, a connectivity term that forces superpixels to form connected entities is introduced. This can be done by penalizing the label, which will break the connectivity of any superpixels. Then, the connectivity term $E_{cnct}\left(x_{i},l_{x_{i}}\right)$ is defined as:

(16) $$E_{cnct}\left(x_{i},l_{x_{i}}\right)=\left\{\begin{array}{c} -\infty \;\;\;\;\;if\;l_{x_{i}}\;break\;connectivity\\ 0\;\;\;\;\;\;\;\;\;otherwise \end{array}\right.$$

### 3.3. Energy Function Optimization

Given a partition ω with the label of each pixel fixed, we can rewrite the energy function Equation (3) as:

(17) $$\begin{array}{c} E\left(\omega \right)=\lambda_{color}\sum_{x_{i}\in I}E_{color}\left(x_{i},l_{x_{i}}\right)+\lambda_{dist}\sum_{x_{i}\in I}E_{dist}\left(x_{i},l_{x_{i}}\right)\\ \;\;\;\;\;\;\;\;\;\;\;+\;\lambda_{smooth}\sum_{x_{i}\in I}E_{smooth}\left(x_{i},l_{x_{i}}\right)+\sum_{x_{i}\in I}E_{cnct}\left(x_{i},l_{x_{i}}\right)\\ \;\;\;\;\;\;\;\;\;=\sum_{x_{i}\in I}(\lambda_{color}E_{color}\left(x_{i},l_{x_{i}}\right)+\lambda_{dist}E_{dist}\left(x_{i},l_{x_{i}}\right)\\ \;\;\;\;\;\;\;\;\;\;\;+\;\lambda_{smooth}E_{smooth}\left(x_{i},l_{x_{i}}\right)+E_{cnct}\left(x_{i},l_{x_{i}}\right)\\ \;\;\;\;\;\;\;\;\;=\sum_{x_{i}\in I}E_{p}\left(x_{i},l_{x_{i}}\right) \end{array}$$

where $E_{p}\left(x_{i},l_{x_{i}}\right)$ is the energy function of pixel $x_{i}$.

According to $E_{p}\left(x_{i},l_{x_{i}}\right)$, the energy value of each pixel is dependent on its surrounding pixels. Hence, it is challenging to construct an optimization algorithm that could achieve the optimal solution. In addition, the cardinalities of ω are huge, which makes it time-consuming to exhaust all of the possibilities for the optimal $\hat{\omega }$. However, superpixel segmentation is time sensitive since it is normally used for image pre-processing. Therefore, we need to make a tradeoff between finding the optimization solution and achieving time efficiency.

To resolve this conflict, we adopt a greedy optimization strategy similar to EM (Expectation Maximization Algorithm) to approximate the optimal solution. Generally, a pixel can only belong to a limited number of superpixels within a restricted local region (as mention in SLIC). Thus, we treat superpixel segmentation as multi-superpixels (for pixel $x_{i}$, multi-superpixels refer to all superpixels in the corresponding set $\Upsilon_{x_{i}}$) competing to obtain pixels. That is to say, for a given pixel $x_{i}$, find the superpixel $R_{n_{max}}$ from the corresponding set $\Upsilon_{x_{i}}$ that maximizing the energy function, as shown in Equation (18). Pixel $x_{i}$ belongs to that superpixel $R_{n_{max}}$, which means it is labeled as $n_{max}$.

(18) $$R_{n_{max}}=\arg\max_{R_{n}\in \Upsilon_{x_{i}}}E_{p}\left(x_{i},n\right)$$

Thus, we optimize the energy function of each pixel iteratively using the following two steps:

1. Given a pixel $x_{i}$, find the label $n_{max}$ that maximizes its energy function $E_{p}\left(x_{i},l_{x_{i}}\right)$ under the current partition ω;
2. Update ω by changing the label of $x_{i}$ to $n_{max}$, i.e., $l_{x_{i}}=n_{max}$.

The two steps repeat iteratively until no energy value changes occur.

## 4. Superpixel Segmentation with Local Competition

According to the optimization strategy described in Section 3.3, a pixel cannot belong to another superpixel when it is inside a superpixel (neighborhood pixels have the same label), because this breaks connectivity and generates an infinitesimal energy value. Hence, only the boundary pixels of superpixels need to be handled when optimizing the energy function. Consequently, the computational complexity is significantly decreased. As a pixel can only belong to superpixels to which its neighboring pixels belong, the superpixel set $\Upsilon_{x_{i}}$, to which pixel $x_{i}$ may belong, could be defined as $\Upsilon_{x_{i}}=\left\{R_{l_{x_{j}}}|x_{j}\in \left(\delta_{x_{i}}^{4}\cup x_{i}\right)\right\}$.

Based on the above analysis, our method includes two main steps: (1) generate initial complete superpixel partitions; (2) iteratively optimize superpixels by utilizing local competition mechanism and, thus, generate superpixels with a coherent appearance that adhere well to image boundaries.

### 4.1. Initialization

The energy function optimization strategy we adopted has an inherent shortcoming in that it may fall into the local optimum solution. Thus, the initial state affects the final result. SPixel and Seeds start from complete superpixel partitions, which are regular lattice grids, and then adopt an optimization scheme to optimize the object function iteratively. Generally, the initial partition is far from the optimal solution, which causes that the final result can easily converge to a local optimal solution.

**Figure 4:**
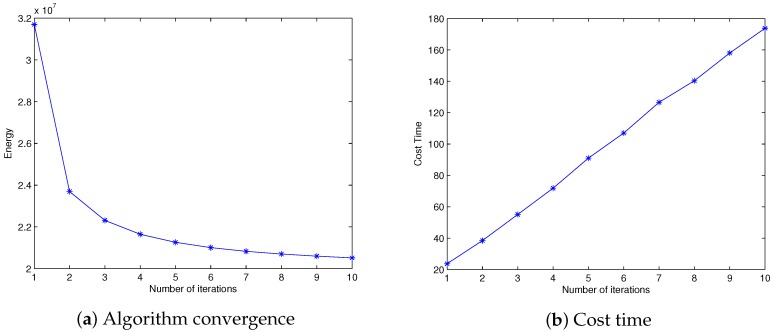
Energy value and cost time of simple linear iterative clustering (SLIC) under different iterations where the specified superpixel number K=300. (**a**) The overall energy value decreases with the increase in iterations; (**b**) the cost time of the main process (k-means cluster and post-processing) increases approximately linearly with each iteration.

SLIC is fast and offers flexibility in the compactness and number of the superpixels it generates [26]. Besides these advantages, we find that its energy value decreases sharply during the first few iterations (as shown in Figure 4a), generating compact, nearly homogenous superpixels after several iterations. From Figure 4b, it can be observed that the time cost of the main process increases approximately linearly with each iteration. Thus, we use a specific version of SLIC (where we set the iteration number to four for efficiency and set compactness to 20 instead of the default value 10 in order to produce more compact and regular regions) called fast SLIC to produce initial partitions. This could reduce the processing time while maintaining performance comparable to the default SLIC.

The initial partitions generated by fast SLIC are more homogenous than lattice grids. They could provide better PDF estimates and center positions for initial superpixels. Hence, the final result could converge to a more optimal solution.

### 4.2. Superpixel Optimization via Local Competition Mechanism

In our optimization scheme, we iterative finding the label for each boundary pixel that maximizes its energy function, as shown in Algorithm 1.

**Algorithm 1** LocalCompetitionSuperpixel**Input:**
ωGeneratedbyfastSLIC**Output:**
ω  1:B= GetBoundaries(ω);  2:**for all** $x_{i}\in B$ **do**  3:    $Indictor[x_{i}]=0$;  4:**end for**  5:**while** B≠∅ **do**  6:    $x_{i}$ = PopFront(*B*);  7:    $E_{max}=0;$  8:    $\Upsilon_{x_{i}}=$ GetNeighborSuperpixelSet($x_{i}$);  9:    **for all** $R_{n}\in \Upsilon_{x_{i}}$ **do**10:        $E_{i}^{n}=ComputeEnergyFunction\left(x_{i},R_{n}\right)$;11:        **if** $E_{max}<E_{i}^{n}$ **then**12:           $E_{max}=E_{i}^{n}$;13:           $n_{max}=n$;14:        **end if**15:    **end for**16:    **if** $l_{x_{i}}\neq n_{max}$ **then**17:        ModifyPDF($R_{n_{max}},x_{i},Add$);18:        ModifyPDF($R_{l_{x_{i}}},x_{i},Remove$);19:        $\delta_{x_{i}}^{4}=GetNeighborPixels\left(x_{i}\right)$;20:        **for all** $x_{j}\in \delta_{x_{i}}^{4}$ **do**21:           **if** $l_{x_{j}}\neq n_{max}$ **then**22:               PushBack($B,x_{j}$);23:               $Indictor[x_{j}]=0$;24:           **end if**25:        **end for**26:        $l_{x_{i}}=n_{max}$;27:        $Indictor[x_{i}]=0$;28:    **else**29:        $Indictor\left[x_{i}\right]=Indictor\left[x_{i}\right]+1$;30:    **end if**31:    **if** $Indictor\left[x_{i}\right]<U_{T}$ **then**32:        PushBack($B,x_{i}$);33:    **end if**34:**end while**

Firstly, we initial active boundary pixels set *B* from the initial partition, which is generated in Section 4.1:

$$B=\left\{x_{i}|l_{x_{i}}\neq l_{x_{j}},x_{j}\in \delta_{x_{i}}^{4}\right\}$$

For each active boundary pixel, we initialize its unchanged indicator to zero.

Then, we iteratively optimize the label for each active boundary pixel from left to right, top to bottom based on maximizing its energy function $E_{p}\left(x_{i},l_{x_{i}}\right)$ as follows:

Given a pixel $x_{i}$, let $l_{before}$ denote its label before optimization and $l_{after}$ denote its label after optimization. When it is observed that $l_{before}$ and $l_{after}$ are identical, this means that the largest energy value is produced when $x_{i}$ is assigned to the current label. Correspondingly, we increase the unchanged indicator of candidate $x_{i}$. If the unchanged indicator is larger than a certain threshold $U_{t}$ ($U_{t}=3$ in the experiment), we call $x_{i}$ a stable boundary pixel. It is reasonable to assume that the label of $x_{i}$, as well as $x_{i}$’s adjacent pixel will not change next time. Hence, we remove $x_{i}$ from the active boundary pixels set *B* for further reduction of computation.

If the label of $x_{i}$ changes, that is $l_{before}\neq l_{after}$, this means $x_{i}$ needing to be moved from $R_{l_{before}}$ to $R_{l_{after}}$. Therefore, the color PDF of the two involved superpixels (the one $R_{l_{before}}$ that $x_{i}$ belongs to before optimization, as well as the one $R_{l_{after}}$ that $x_{i}$ belongs to after optimization) needs to be updated. The update will be done as follows: given the previously estimated $P_{R_{k}}\left(c\right)$, find clusters $S_{m}$ to which $c_{x_{i}}$ belongs according to the Euclidean distance, and then, update multivariate polynomials coefficient of that cluster. Besides that, the new label changes the boundary of $R_{l_{before}}$ and $R_{l_{after}}$. Hence, for each neighbor pixel $x_{j}$, $x_{j}\in \delta_{x_{i}}^{4}$, we check whether the label of $x_{j}$ equals $l_{after}$. We call $x_{j}$ the active boundary pixel if its label is not equal to $l_{after}$, that is $l_{x_{j}}\neq l_{after}$. Then, we push $x_{j}$ into the active boundary pixels set *B* and initialize its unchanged indicator to zero.

The iteration will continue until *B* is empty, that is until all boundary pixels are stable and the energy function of the boundary pixels is maximized.

## 5. Experiments

The proposed superpixel algorithm SSLC is implemented in C++ and tested on a laptop with an Intel I5-3230M (2.60 GHz) CPU and an 8GB RAM. SSLC is compared with eleven state-of-the-art superpixel algorithms including SLIC [26], SPixel [35], Seeds [37], FH [40], QS [41], TurboPixel [42], TPS [45], LSC [46], MSLIC [47], ERS [25], and ERGC [52] on the entire Berkeley Segmentation Dataset [38] (BSD), which includes five hundred images with multiple ground truth contours and segmentations. For all of the eleven state-of-the-art algorithms, the implementations are based on publicly-available code, and we used the default parameter settings provided by the authors for achieving as fair comparison as possible.

### 5.1. Quantitative Comparison

We quantitatively evaluated the quality of these superpixel algorithms mainly by using three commonly-used evaluation metrics in image segmentation: Boundary Recall (BR), Under-segmentation Error (UE) and Achievable Segmentation Accuracy (ASA). The comparison results are obtained by averaging these metrics across all images and all ground truth segments in the BSD benchmark.

For the sake of clarity, our segmentation is defined as $S=\left\{R_{1},R_{2},\ldots ,R_{n_{s}}\right\}$, and the ground truth is defined as $G=\left\{G_{1},G_{2},\ldots ,G_{n_{g}}\right\}$; $n_{s}$ and $n_{g}$ are the number of segments in each set. The boundaries of superpixels and ground truth segments are expressed as δS and δG.

BR measures the fraction of ground truth boundaries correctly recovered by the superpixel boundaries. A true boundary pixel is regarded to be correctly recovered if it falls within ε pixels from at least one superpixel boundary pixel. Then, the BR is defined as:

(19) $$BR\left(S,G\right)=\frac{\sum_{x_{i}\in \delta G}[\;[\left(\underset{x_{j}\in \delta S}{\min∥u_{x_{i}}-u_{x_{j}}∥<\varepsilon }\right)}{\left|\delta G\right|}$$

The indicator function [[ checks whether the nearest pixel is within ε (ε=2 in these experiments) distance. A high BR value indicates that very few true edges are missed.

UE measures the percentage of pixels that leak from the ground truth boundaries. It actually evaluates the quality of superpixel segmentation by penalizing superpixels overlapping with multiple objects. Formally, the UE is quantified as:

(20) $$UE\left(S,G\right)=\frac{1}{N}\left[\sum_{i=1}^{n_{g}}\left(\sum_{R_{j}|R_{j}\cap G_{i}>\kappa }|R_{j}|\right)-N\right]$$

κ is set to 5% of $R_{j}$ to account for ambiguities in the ground truth. The smaller the under-segmentation error, the better the precision with which the superpixels overlap with only one object.

ASA is defined as the highest achievable object segmentation accuracy when utilizing superpixels as units. By labeling each superpixel with the ground truth segments of the largest overlapping area, ASA is calculated as the fraction of labeled pixels that are not leaked from the ground truth boundaries. Mathematically, ASA is computed as follows:

(21) $$ASA\left(S,G\right)=\frac{\sum_{k}\max_{i}\left|R_{k}\cap G_{i}\right|}{\sum_{i}\left|G_{i}\right|}$$

**Figure 5:**
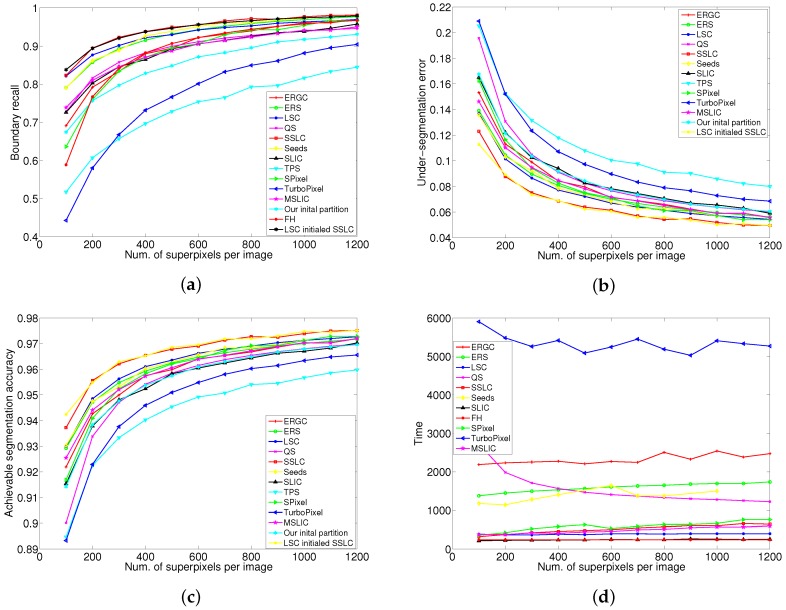
Performance vs. number of superpixels on the Berkeley Segmentation Dataset (BSD) benchmark. Higher Boundary Recall (BR) and Achievable Segmentation Accuracy (ASA) values indicate a better performance in superpixel alignment; lower Under-segmentation error (UE) values represent less error in the superpixel result. (**a**) Boundary recall; (**b**) Under-segmentation error; (**c**) Achievable segmentation accuracy; (**d**) Time.

The BR performance metric is plotted as a function of the number of superpixels in Figure 5a. From the plots, our algorithm yields the best recall ratio across the entire range of superpixel counts. LSC (an extended SLIC algorithm) and the proposed algorithm SSLC have an approximately equal recall ratio with 100 superpixels. However, for a larger number of superpixels, SSLC significantly outperforms LSC. Seeds (which also uses the boundary optimization scheme) and ERS show a slightly poorer recall ratio than SSLC and LSC on small numbers of superpixels. However, their recall ratio gradually approximates that of SSLC as the number of superpixels increases. Moreover, those two algorithm are superior to LSC when the number of superpixels increases greatly. As can be seen, the above four algorithms significantly outperform the other tested ones, especially when the numbers of superpixels is small.

Figure 5b shows the comparison results of UE. As FH produces superpixels with highly irregular shapes and sizes, its performance is quite poor in UE and therefore omitted in Figure 5b for clarity. From the above graph, it can be seen that SSLC outperforms all other tested algorithms by a significant gap. LSC and Seeds have a similar error rate across the entire range of superpixel counts. The error rate of ERS is quite close to that of LSC when the number of superpixels is small. However, in the case of a larger number of superpixels, it marginally underperforms LSC. The error rates of ERS and MSLIC (an extended SLIC algorithm) remain quite close and underperform that of the above algorithms. SPixel (which also uses the boundary optimization scheme) shows a relatively poor error rate with a small number of superpixels. However, as the number of superpixels increases, its error rate improves quickly. SLIC and QS show worse performance than the above algorithms. Turbopixel and TPS show the worst performance among the tested algorithms.

According to SLIC [26], adherence to image boundaries is the most important property for superpixel algorithms, and the standard measures for boundary adherence are UE and BR. From the plot in Figure 5a,b, the proposed algorithm SSLC achieves the highest BR and least UE, which shows its superior performance in boundary adherence among all tested algorithms.

Figure 5c plots the ASA performance curves for all tested algorithms (the performance of FH is rather poor and therefore omitted in Figure 5c). As can be seen, SSLC outperforms all other tested algorithms by a significant gap. This means that the proposed algorithm could yield a much better achievable segmentation upper-bound.

From Figure 5, it can be observed that the proposed algorithm significantly outperforms our initial partitions. For example, when the number of superpixels is 100, the proposed algorithm improves the BR from 0.67 to 0.82, and reduces the UE from 0.17 to 0.12, as well as improves the ASA from 0.91 to 0.94. This shows that the proposed local competition mechanism significantly boosts performance on the initial partition. Nevertheless, the proposed algorithm is not restricted to using the initial partition, which is generated by fast SLIC. It can also start from any other complete partition, such as LSC. As can be seen in Figure 5, the performance of LSC-initialized SSLC also outperform LSC. The impact of initial partition will be discussed in Section 5.3.

Another important aspect of superpixel algorithms is computational efficiency. Since superpixels are often used as a preprocessing step, the processing time is an important factor, such that it does not slow down the image processing pipeline. In the experiment, we calculated the average running time for all tested algorithms, and the results are shown in Figure 5d. TPS requires about 26 seconds to over-segment an image, which is much higher than that of the other algorithms. Thereby, TPS is not plotted due to its particularly slow speed. From the curves, it can be observed that SLIC and FH run fastest among all of the tested algorithms. The runtime performance of LSC marginally underperforms that of the above two algorithms and is independent of the number of superpixels. The proposed algorithm slightly outperforms LSC with 100 superpixels, but it increases approximately linearly with respect to the number of superpixels. This can be explained as the more superpixels there are, the more boundary pixels will be generated, leading to more boundary optimization iterations. The runtime performance of MSLIC is similar to ours and slightly outperforms that of SPixel. The remaining algorithms show a significant gap in computational efficiency.

### 5.2. Qualitative Comparison

**Figure 6:**
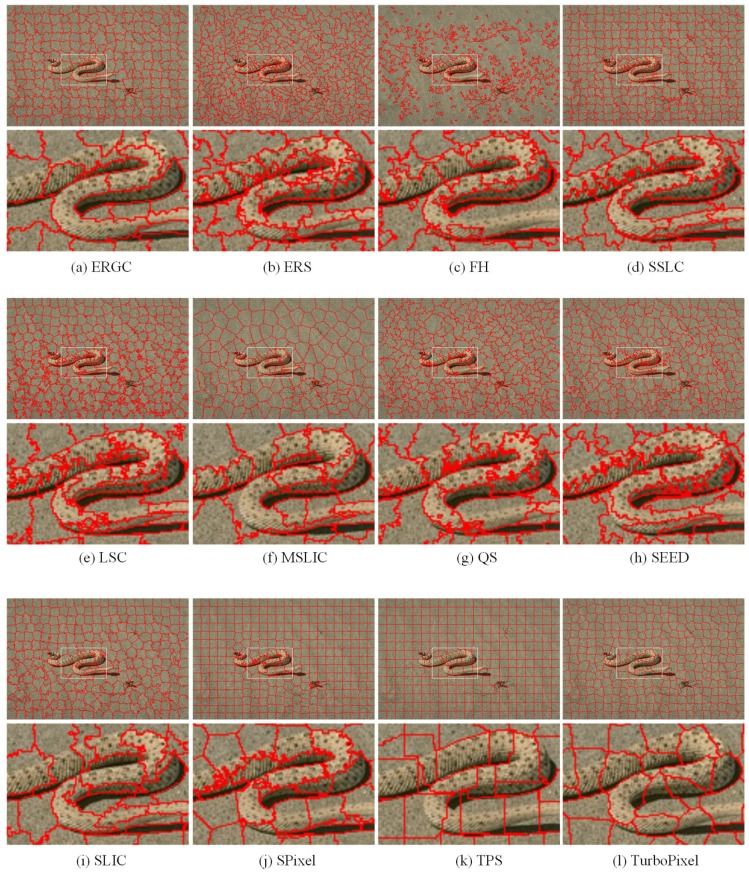
Superpixel segmentations from an image with low variance in color. The first, third and fifth rows are superpixels generated by various methods. The second, fourth and sixth rows zoom in on the regions of interest as defined by the white boxes to facilitate close visual inspection.

**Figure 7:**
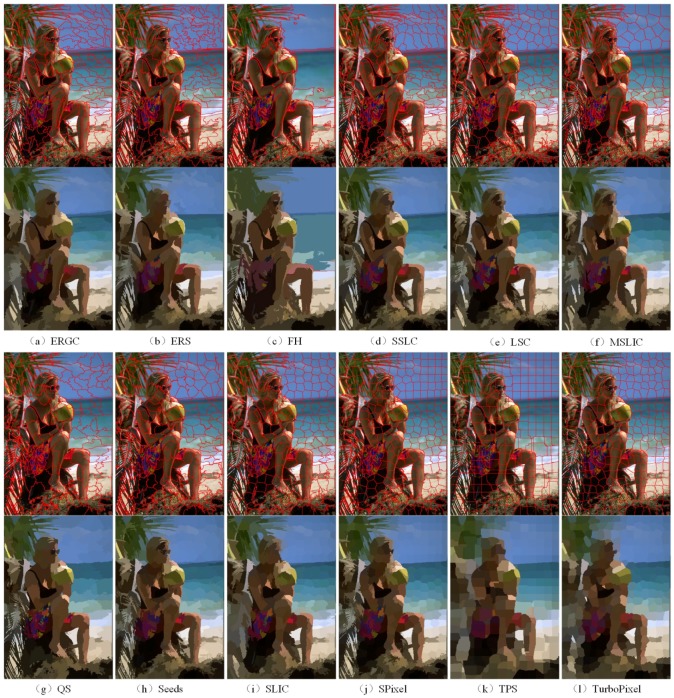
Superpixels obtained by all tested superpixel algorithms in an example image with heterogeneous contents and scene layouts. The top and third rows are the results of various superpixel algorithms, and the second and bottom rows are region mean images corresponding to the superpixel segmentations in the top and third rows.

For qualitative evaluation, Figure 6 and Figure 7 show superpixels obtained by all tested superpixel algorithms in the two images where the number of superpixels is 300. It can be seen that those two images have a significant difference in image content and scene layout.

Figure 6 is superpixels generated from an image with low color variation. We can see that color and texture differences are small between the snake and the background. Moreover, the contours of the snake are blurred. This leads to superpixels algorithms that are based on gradient, such as TurboPixel and ERGC, missing most of the contours of the snake. TPS, which is based on boundary maps, also shows poor performance in boundary recovery. SLIC, MSLIC and LSC are based on iterative k-means clustering. They show better performance in capturing the contours of the snake, but still miss partial contours obviously. SPixel and Seeds also utilize the boundary optimization scheme. However, their energy terms are insensitive to small differences, leading those two algorithms to produce a poor boundary adherence result. Visually, superpixels obtained by the proposed algorithm snap to the contours of the snake very well and only miss several boundary pixels. This is mainly because our energy terms are relative membership degrees, which are generated by the local competition mechanism. Therefore, the proposed algorithm could differentiate these small differences and produces superpixels that snap well to the contours of the snake.

For the sake of completeness, Figure 7 shows superpixel segmentations using an image with heterogeneous scene layouts. The superpixels, generated by TurboPixel and TPS, have a regular shape and size. However, they show poor performance in boundary adherence, which causes that the mean image not to be able to distinguish the contours of the women. The superpixels obtained by FH adhere well to image boundaries in most cases. However, their shape and size are very irregular, leading to relatively poor segmentation performance. As can be seen in Figure 7c, FH segments hair and leaves in one region incorrectly. The clustering-based methods, SLIC, MSLIC and LSC, can obtain the contours of the people, whereas they show poor boundary adherence in regions that contain prominent boundaries (such as the palm leaves region in Figure 7e,f,i). Superpixel boundaries of SPixel and Seeds react to prominent boundaries better. However, in regions with high color variation, those two algorithms show poor performance (such as the skirt regions in Figure 7h,j). Intuitively, the proposed algorithm achieves the most perceptually satisfactory segmentation results. It extracts the most details precisely, such as skirt, T-shirt sling, hair and green leaves, etc. This is because of that the proposed energy function and energy terms locality and relativity. As a result, our algorithm is less sensitive to the changes of content and scene layout.

Overall, the proposed algorithm shows excellent performance in both sample images despite the color, intensity variation and layout changing greatly. This demonstrates that the proposed algorithm, which is based on the local competition mechanism, could achieve consistent performance over diverse image contents and scene layouts.

### 5.3. Discussion of Initial Partitioning

**Figure 8:**
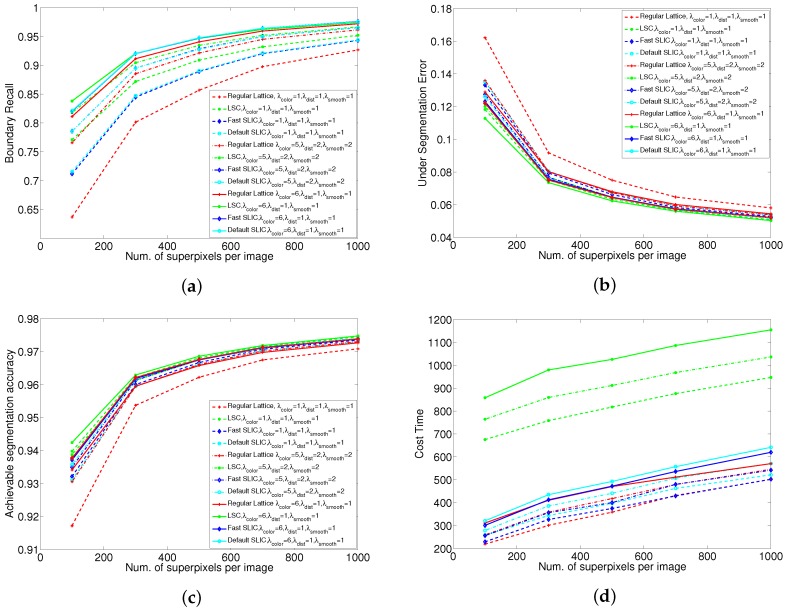
Performance and efficiency comparison of our method under different initial partitioning. (**a**) Boundary recall; (**b**) Under-segmentation error; (**c**) Achievable segmentation accuracy; (**d**) Efficiency.

The influence of different initial superpixel partitions are shown in Figure 8. From Figure 8a, it can be observed that the performance of LSC-initialized SSLC yields the best overall performance especially on small numbers of superpixels. And it also can be seen that the proposed algorithm starting from fast SLIC is much better than from regular lattice under any weight configurations. This indicates that the better initial partitioning could improve the performance of the proposed Algorithm.

From Figure 8, it can be seen that when $\lambda_{color}$ is relatively small, the initial partition yields larger influence on the final performance. However, the larger the value of $\lambda_{color}$, the smaller the influence that the initial partition yields. When we set the weight of energy terms as $\lambda_{color}=6,\lambda_{dist}=1,\lambda_{smooth}=1$, the influence of different initial partitions have almost no difference especially on large numbers of superpixels. Even though we use a regular lattice grid as the initial partition, the recall rates are 81% and 94% with 100 and 500 superpixels, respectively. The recall rates with the same superpixel counts are 82% and 93% by LSC. Therefore, the performance of regular lattice initialized SSLC still compares favorably to state-of-the-art superpixel algorithms. This indicates that the proposed algorithm is not restricted to a specified initial superpixel partition.

Judging the results on the three standard metrics from Figure 8a–c, it can be observed that the BR, UE and ASA performances show almost no difference between the default SLIC and the fast SLIC. This is because after k-means clusters four-times in SLIC, each superpixel is nearly coherence and has little difference with the results of default SLIC, satisfying our algorithms’s requirement for initial segmentation conditions. From Figure 8d, it can be observed that the proposed algorithm costs more time if we use default SLIC as the initial partitioning rather than fast SLIC. As initial partitions based on fast SLIC could improve efficiency yet still maintain performance, therefore the fast SLIC is a good alternative method for initial superpixel partitions.

### 5.4. Energy Terms Analysis

The proposed algorithm use four energy terms, among which the appearance coherence term and the shape regularization term play the major role, to construct the energy function. Other than most state-of-the-art superpixel algorithms, we adopt more sophisticated measure to construct these two major energy terms. We adopt KDE-estimated PDF instead of mean value and histogram to construct the appearance coherence term, and adopt geodesic distance instead of Euclidean distance to construct the shape regularization term.

**Figure 9:**
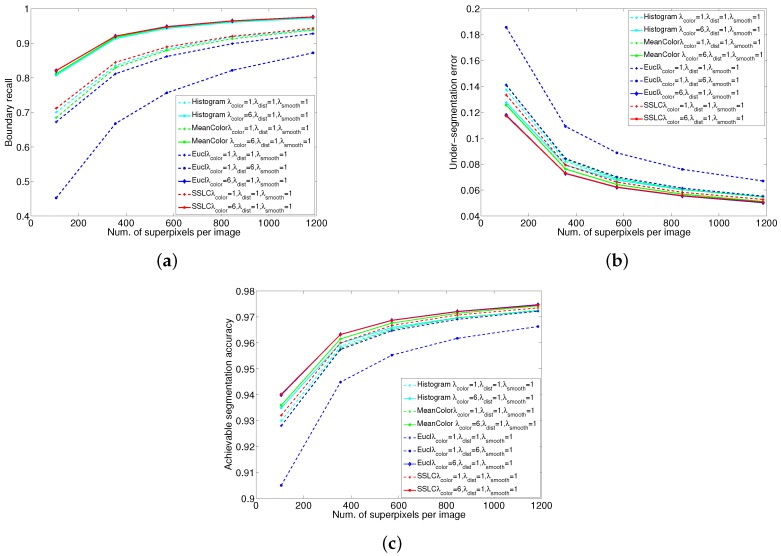
Performance comparisons of the proposed algorithm with different energy terms. (**a**) Boundary recall; (**b**) Under-segmentation error; (**c**) Achievable segmentation accuracy.

From Figure 9, it can be observed that when setting equal weights for all the energy terms. Histogram-based SSLC yields better performance than mean color-based SSLC. This is because of that histogram describe the color distribution of superpixels more sophisticated than mean color. Compared with Euclidean distance, geodesic distance not only promotes the generated superpixels compactness, but also promotes superpixels adhering to image boundaries. Therefore, Euclidean distance-based SSLC significantly underperforms SSLC in BR. The proposed algorithm adopts sophisticated measure to construct both the appearance coherence term and the shape regularization term, and thus yields the best performance.

### 5.5. Parameter Analysis

**Figure 10:**
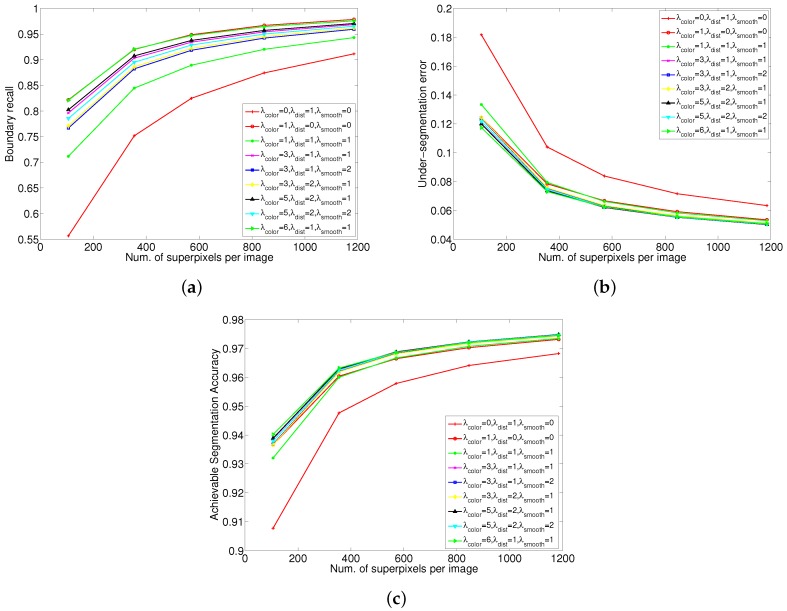
Performance comparison of the proposed algorithm under different parameter configurations. (**a**) Boundary recall; (**b**) Under-segmentation Error; (**c**) Achievable segmentation accuracy.

**Figure 11:**
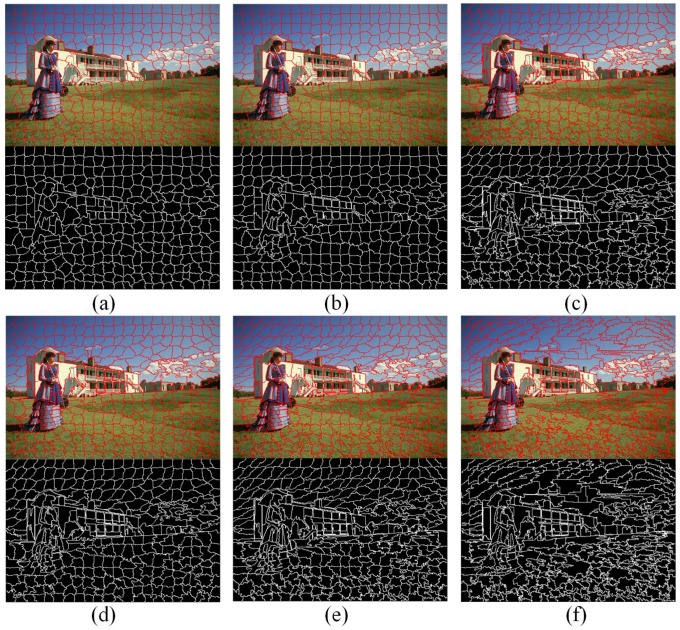
Typical visual results show the impact of different energy terms in the energy function. (**a**) is a shape regularization term only $\left(\lambda_{color}=0,\lambda_{dist}=1,\lambda_{smooth}=0\right)$; (**b**) is different terms with equal weights $\left(\lambda_{color}=1,\lambda_{dist}=1,\lambda_{smooth}=1\right)$; (**c**) weights of different energy terms are $\left(\lambda_{color}\;=\;5,\lambda_{dist}\;=\;2,\lambda_{smooth}=1\right)$; (**d**) weights of different energy terms are $\left(\lambda_{color}\;=\;3,\lambda_{dist}\;=\;1,\lambda_{smooth}\;=\;1\right)$; (**e**) weights of different energy terms are $\left(\lambda_{color}\;=\;6,\lambda_{dist}\;=\;1,\lambda_{smooth}=1\right)$; and (**f**) is a color appearance term only $\left(\lambda_{color}\;=\;1,\lambda_{dist}\;=\;0,\lambda_{smooth}=0\right)$.

The proposed algorithm uses three parameters $\lambda_{color}$, $\lambda_{dist}$, $\lambda_{smooth}$ to control the relative significance of appearance coherence and shape regularity. Figure 10 show the performance of the proposed algorithm under difference parameter configurations. From Figure 10a, it can be seen that relatively larger $\lambda_{color}$ results in generated superpixels with better BR. If we only use a shape regularization term, it produces superpixels with the worst BR performance. However, if we only use a color appearance term, it performs the best. From Figure 10b,c, the configuration that only use a appearance coherence term show worse UE and ASA than most of other parameter configurations. This is because of that without compactness constraint, such algorithms produce superpixels with irregular shapes and sizes. The size of a superpixel may be very large in regions absence of boundary cues, and if it overlap more than one object, it will lead to the UE and ASA greatly reduced. In fact, there is a trade-off among different energy terms. Figure 11 visualizes the effect of different weight configurations on the resulting superpixels. It can be observed that relatively larger $\lambda_{color}$ results in more irregular shapes, but shows better boundary adherence.

For some applications, boundary adherence is the most important property for superpixels; whereas for some other applications, more regular shapes may be preferable, such as in [29,30]. Hence, for most of superpixel algorithms, such as [26,35,37], etc., the ability to control this tradeoff is their important property. From Figure 10 and Figure 11, it can be seen that the proposed algorithm also control this tradeoff well.

### 5.6. Evaluation on Remote Sensing Images

Three HSIs are utilized to conduct experiments to evaluate the performance of SSLC on remote sensing images. The first two images are Indian Pines and Salinas, which were gathered by the Airborne Visible Infrared Imaging Spectrometer (AVIRIS) sensor. Those two images mainly consist of large-sized homogeneous regions. The Indian Pines image has 220 bands of size 145×145 pixels, and the Salinas image has 224 bands of size 512×217 pixels. The other image PaviaU was acquired by the Reflective Optics System Imaging Spectrometer (ROSIS) sensor, which consists of more detailed structures. The PaviaU image has 103 spectral bands, and the size is 610×340 pixels.

**Figure 12:**
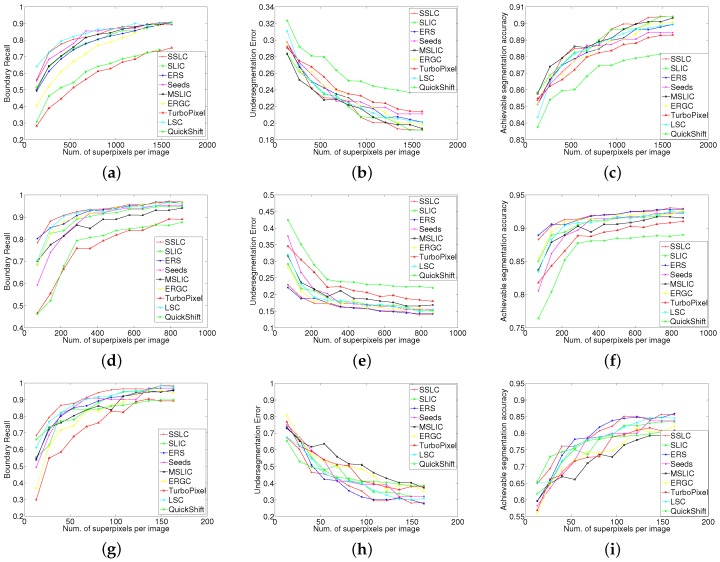
Performance comparison of the proposed algorithm and the other tested ones on remote sensing images. (**a**) PaviaU BR; (**b**) PaviaU UE; (**c**) PaviaU ASA; (**d**) Salinas BR; (**e**) Salinas UE; (**f**) Salinas ASA; (**g**) Indian Pines BR; (**h**) Indian Pines UE; (**i**) Indian Pines ASA.

The HSIs have too many bands; thereby, it is time costly to over-segment the original HSIs into superpixels. It also is not able to excavate spatial information in HSIs if just one band is selected for superpixel segmentation. Therefore, in the papers [3,6,9,10,11,19], they applied the Principal Component Analysis (PCA) algorithm on the original HSIs to reduce the computational cost. Since the first Principal Component (PC) contains the major information of the HSIs, they denote it as a fundamental image. Similar to those methods, we also utilize the first PC to generate the base image. Then, the standard SLIC, SSLC, ERS, Seeds, MSLIC, ERGC, TurboPixel, LSC and QS were performed separately on the base images. The quantitative comparison results are shown in Figure 12.

From Figure 12, it can be seen that in scene PaviaU, the BR curves generated by Seeds and the proposed algorithm cross each other, and the latter has a larger maximum value than the former. LSC outperform those two algorithms when the number of superpixels is small. In the case of a larger number of superpixels, the recall ratio of LSC is quite close to that of the proposed algorithm. However, LSC shows worse performance on UE and ASA than the proposed algorithm. In scene Salinas, the proposed algorithm achieves the best BR, UE and ASA in most case. In the scene Indian Pines, the proposed algorithm outperforms other tested superpixel algorithms on BR with a significant gap on small numbers of superpixels. The proposed algorithm also achieves or approaches the best performance on UE and ASA. By analysis the experiment results, it can be seen that the proposed algorithm yields the best overall performance on HSIs.

**Figure 13:**
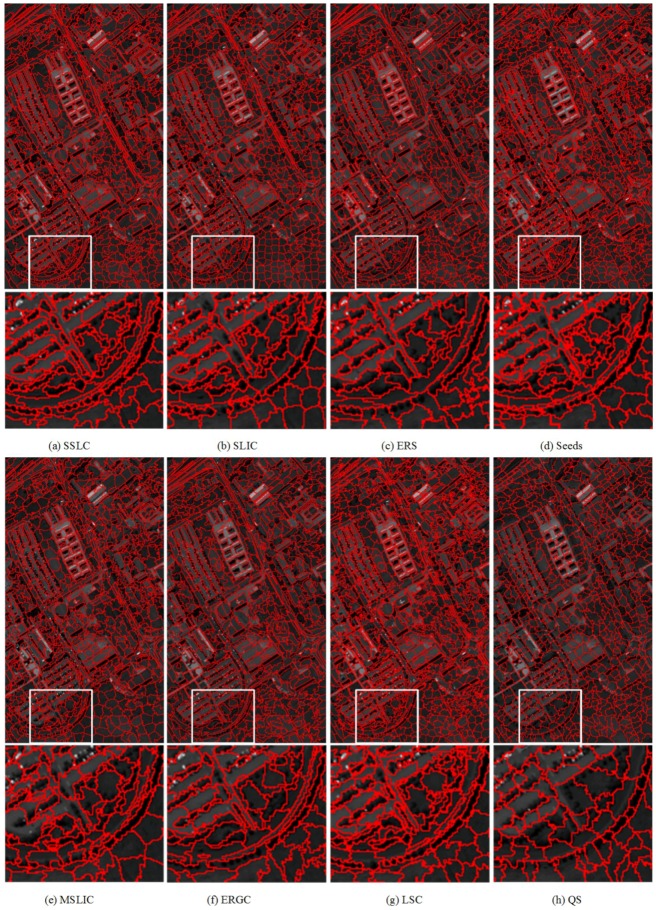
The first and third rows are superpixel segmentations of the Reflective Optics System Imaging Spectrometer (ROSIS) image PaviaU provided by: (**a**) Superpixel Segmentation with Local Competition (SSLC); (**b**) SLIC; (**c**) Entropy Rate Superpixel (ERS); (**d**) Seeds; (**e**) MSLC; (**f**) ERGC; (**g**) Linear Spectral Clustering (LSC); and (**h**) Quick Shift (QS). The second and fourth rows zoom in on the regions of interest as defined by the white boxes to facilitate close visual inspection.

**Figure 14:**
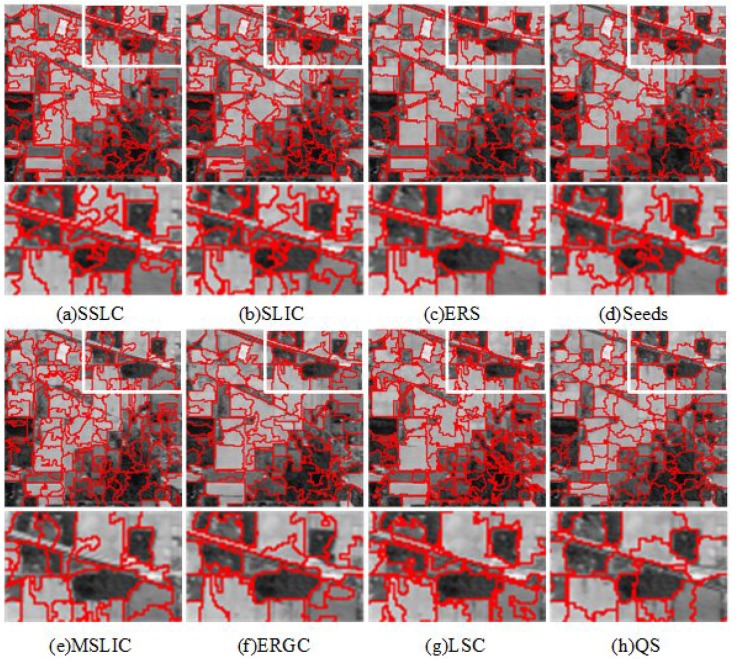
The first and third rows are superpixel segmentations of AVIRIS image Indian Pines provided by (**a**) SSLC; (**b**) SLIC; (**c**) ERS; (**d**) Seeds; (**e**) MSLC; (**f**) ERGC; (**g**) LSC; and (**h**) QS. The second and fourth rows zoom in on the regions of interest as defined by the white boxes to facilitate close visual inspection.

**Figure 15:**
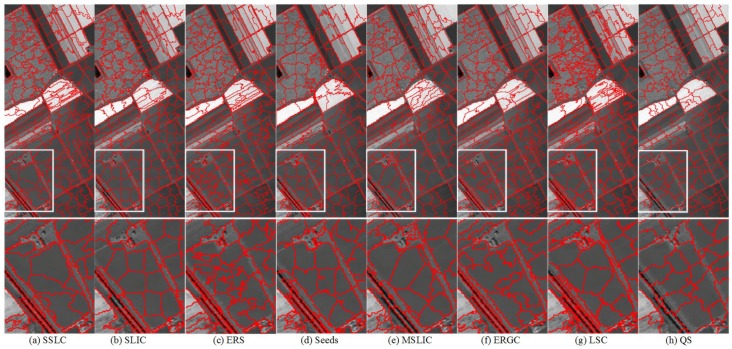
The first row is superpixel segmentations of AVIRIS image Salinas provided by (**a**) SSLC; (**b**) SLIC; (**c**) ERS; (**d**) Seeds; (**e**) MSLC; (**f**) ERGC; (**g**) LSC; and (**h**) QS. The second row zooms in on the regions of interest as defined by the white boxes to facilitate close visual inspection.

To further evaluate the performance of those testing methods for segmenting HSIs into superpixels, some results of representative superpixels are shown in Figure 13, Figure 14 and Figure 15. By comparing the superpixels of the three images, it can be visually observed that the superpixels that are generated by the proposed algorithm yield the best results especially in the white box regions.

## 6. Conclusions

A local competition-based superpixel segmentation algorithm is proposed in this paper. It produces perceptually coherent superpixels with linear time. The most critical idea in SSLC is using a local competition mechanism to construct energy terms and label pixels. Energy terms are membership degrees and therefore relative. This makes the proposed algorithm less sensitive to the changes of image content and scene layout, and it performs consistently across image regions. Additionally, a KDE-estimated PDF is introduced to describe the color distribution of superpixels. This description model achieves a more sophisticated and accurate energy formulation than most state-of-the-art methods. Moreover, a boundary optimization scheme is introduced to deal with and only with the boundary pixels. This scheme significantly decreases the computational complexity.

Experimental results show that the proposed algorithm outperforms state-of-the-art ones with respect to boundary recall and under-segmentation error, which shows its superior performance in covering the perceptually coherent region with an adaptive shape and structure. In most case, the proposed algorithm achieves the highest achievable segmentation accuracy, which means it could yield a much better achievable segmentation upper-bound. Therefore, it can be considered a good alternative to other existing state-of-the-art superpixel algorithms for remote sensing image processing and many other fields.

Our future work is to design a new energy function and an optimization scheme that can produce superpixels while preserving the topology (since topology is critical for some applications). Another aspect is to improve the efficiency of our algorithm for real-time applications.
